# Prediction and prioritization of autism-associated long non-coding RNAs using gene expression and sequence features

**DOI:** 10.1186/s12859-020-03843-5

**Published:** 2020-11-07

**Authors:** Jun Wang, Liangjiang Wang

**Affiliations:** 1 Department of Genetics and Biochemistry, Clemson University, Clemson, SC 29634 USA; 2 Center for Human Genetics, Clemson University, Clemson, SC 29634 USA

**Keywords:** ASD risk genes, lncRNAs, Candidate prioritization, Machine learning, Autoencoder

## Abstract

**Background:**

Autism spectrum disorders (ASD) refer to a range of neurodevelopmental conditions, which are genetically complex and heterogeneous with most of the genetic risk factors also found in the unaffected general population. Although all the currently known ASD risk genes code for proteins, long non-coding RNAs (lncRNAs) as essential regulators of gene expression have been implicated in ASD. Some lncRNAs show altered expression levels in autistic brains, but their roles in ASD pathogenesis are still unclear.

**Results:**

In this study, we have developed a new machine learning approach to predict candidate lncRNAs associated with ASD. Particularly, the knowledge learnt from protein-coding ASD risk genes was transferred to the prediction and prioritization of ASD-associated lncRNAs. Both developmental brain gene expression data and transcript sequence were found to contain relevant information for ASD risk gene prediction. During the pre-training phase of model construction, an autoencoder network was implemented for a representation learning of the gene expression data, and a random-forest-based feature selection was applied to the transcript-sequence-derived *k*-mers. Our models, including logistic regression, support vector machine and random forest, showed robust performance based on tenfold cross-validations as well as candidate prioritization with hypothetical loci. We then utilized the models to predict and prioritize a list of candidate lncRNAs, including some reported to be *cis*-regulators of known ASD risk genes, for further investigation.

**Conclusions:**

Our results suggest that ASD risk genes can be accurately predicted using developmental brain gene expression data and transcript sequence features, and the models may provide useful information for functional characterization of the candidate lncRNAs associated with ASD.

## Background

Autism spectrum disorders (ASD) refer to a broad range of neurodevelopmental conditions characterized by symptoms of having difficulties in social interactions, verbal and non-verbal communications, and showing repetitive behaviors. Autism is genetically heritable, and usually begins in infancy, at the latest, in the first three years of life [1]. The genetic etiology of ASD is complex and highly heterogeneous with almost all genetic risk factors also found in the unaffected general population [2]. According to the Simons Foundation Autism Research Initiative (SFARI) (https://www.sfari.org/resource/sfari-gene/), 913 genes and 17 recurrent copy number variation (CNV) loci have been implicated in ASD, but how these diverse genomic aberrations cause ASD is poorly understood. Currently, all the known ASD risk genes code for proteins, and some de novo mutations that likely disrupt protein-coding genes have been shown to cause ASD [3–5]. However, a recent analysis based on 1,790 ASD simplex families has revealed that the vast majority of de novo mutations are located in non-coding regions and linked with the IQ heterogeneity of ASD probands [3].

Long non-coding RNAs (lncRNAs), defined as transcripts greater than 200 nucleotides and not encoding proteins, are emerging as essential regulators of gene expression [6]. While the human genome expresses a large number of lncRNAs, only some lncRNAs have been functionally characterized with proposed roles in gene regulation at the transcriptional, post-transcriptional, translational, or epigenetic levels [7–9]. LncRNAs can be brain-enriched, and involved in brain development, neuron function and maintenance, and neurodegenerative diseases [10, 11]. Some lncRNAs show altered expression levels in autistic brains [11] and may constitute a new class of candidate genes contributing to ASD. However, the relatively low expression levels of lncRNAs in human cells and lack of protein products pose challenges for the functional characterization of lncRNAs using experimental techniques designed for protein-coding genes. ASD-associated lncRNAs may be identified through differential gene expression analysis, and several lists containing various numbers of lncRNAs have been reported, giving rise to the current situation with many unprioritized candidates for further investigation. Thus, an accurate model for the prediction and prioritization of ASD-associated lncRNAs can be valuable.

For ASD diagnosis, computational prediction models have been developed by using various types of clinical data from ASD patients, such as symptom profiles, magnetic resonance image (MRI) data and whole-brain structural image data [12–16]. However, these models are not applicable for the prediction of ASD risk genes. Genetic approaches for identifying ASD risk genes, such as genome-wide association studies (GWAS), copy number variation studies (CNVs) and whole exome sequencing (WES), are time-consuming and expensive. Recently, by using the brain gene expression profiles in the BrainSpan dataset, our group developed a support vector machine (SVM) model for the prediction and prioritization of ASD-associated candidate lncRNAs [17], as the expression patterns of ASD risk genes are distinct in autistic brains [18, 19]. Although computational models have been reported for predicting potential disease-lncRNA associations [20–25], the applicability to ASD risk genes has not yet been demonstrated.

In this study, to build models for accurate prediction and prioritization of ASD-associated lncRNAs, we have tested various machine learning algorithms, including logistic regression (LR), SVM and random forest (RF). Both developmental brain gene expression profiles and RNA transcript sequence compositions were used as features for model construction. To reduce the high dimensionality of the input features, which might cause model overfitting, an autoencoder network, gene embedding [26], and RF-based feature selection were tested. Lastly, we utilized the models to predict and prioritize ASD-associated candidate lncRNAs, which might provide a good list of targets for further investigation.

## Results

**Fig. 1:**
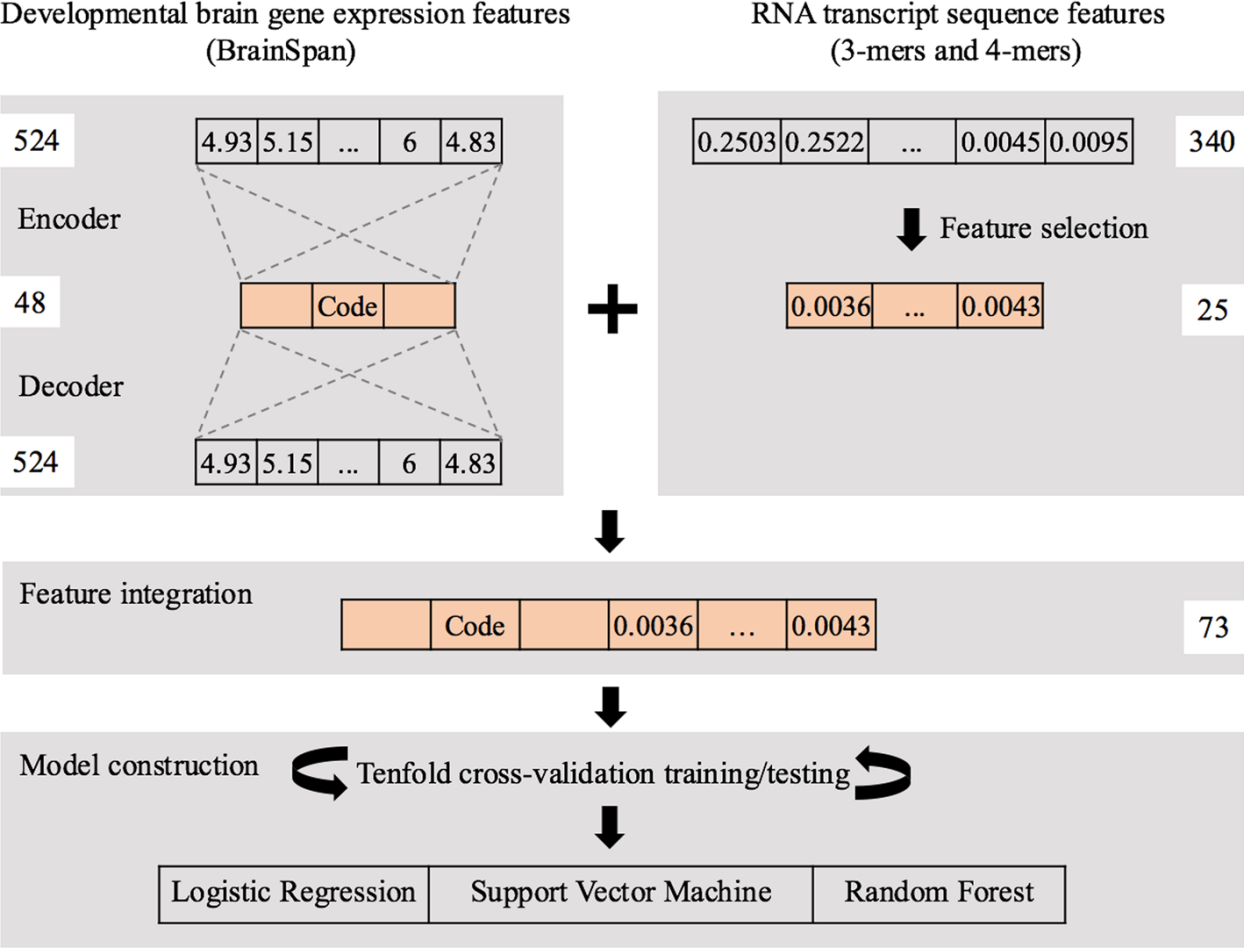
Schematic diagram of model construction. The autoencoder-derived expression features and selected sequence features (*k*-mers) were combined for model construction

In this study, we used both developmental brain gene expression profiles and RNA transcript sequence compositions as features to construct LR, SVM and RF models for ASD risk gene prediction (Fig. 1). To reduce the high dimensionality of input features, an autoencoder network was implemented for the representation learning of gene expression data, and an RF-based method was used for the selection of sequence features important for classification. With the combined gene expression and sequence features, LR, SVM and RF models were trained and evaluated using a tenfold cross-validation strategy.

### Autoencoder-based representation learning of developmental brain gene expression data

**Fig. 2:**
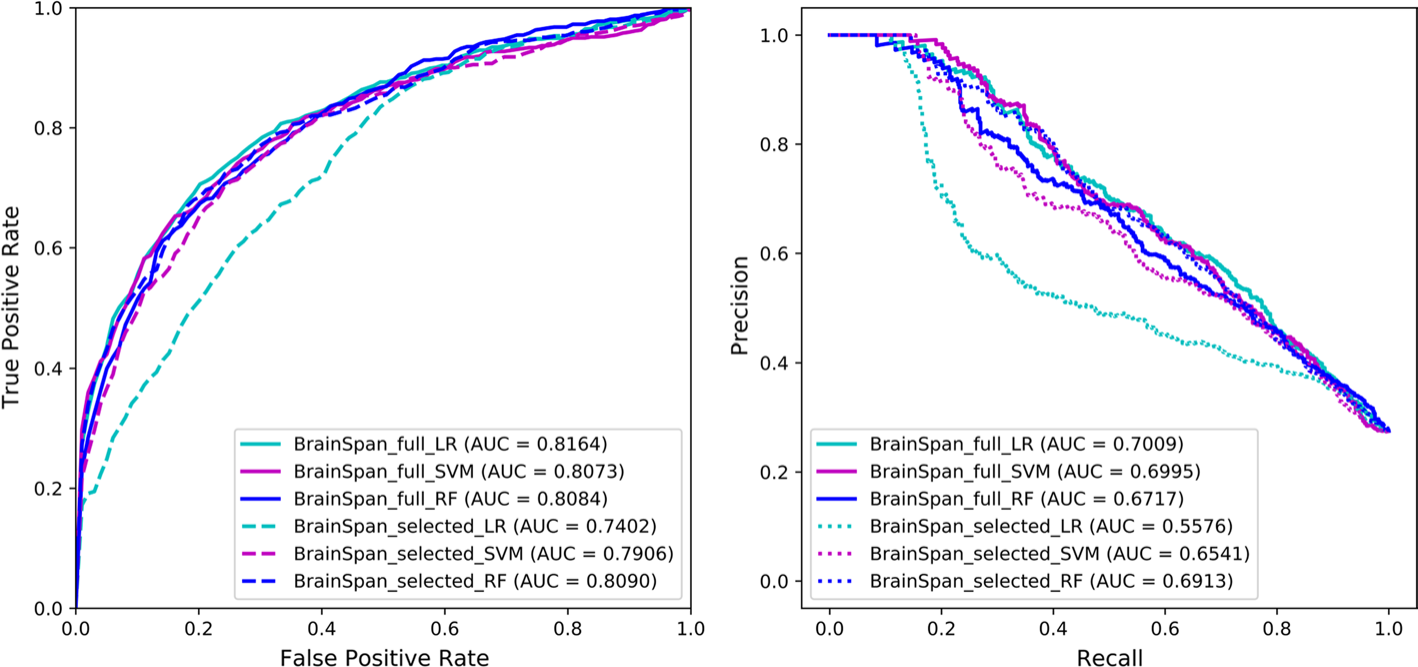
ROC curves (left) and PR curves (right) to compare the models trained using either the full set of 524 expression features or the 15 features selected in a previous study [17]. Models were trained and evaluated using tenfold cross-validations

In a previous study from our group, an SVM model for ASD risk gene prediction was constructed using developmental brain gene expression data from BrainSpan [17]. In this study, with the updated training dataset, we first examined the performance of LR, SVM and RF models using the same set of gene expression features (BrainSpan_full). As shown in Fig. 2, the models showed comparable or slightly improved performance when compared with the previous SVM model [17]. To improve model performance and avoid possible overfitting, we tested several methods to reduce the high dimensionality of gene expression features. In the previous study [17], 15 gene expression features were selected using the wrapper method with best-first heuristic search, and then used to construct an SVM model with slightly improved performance. As each gene expression feature represented one developmental time point of a brain region, the 15 selected features were enriched for early development, particularly from 8 weeks post conception to one year of age [17]. In this study, however, the models showed similar or decreased performance with the 15 selected features when compared with the models using the full set of features (Fig. 2). The inconsistency of model performance with the 15 selected features might be due to the updated training dataset. In the previous study [17], the training dataset consisted of 366 ASD risk genes as positive instances and 1,762 non-ASD disease genes as negative instances. Since many additional genes, including some of the negative instances in the previous dataset, have recently been identified as ASD risk genes, the training dataset in this study includes 604 positive instances and 1,594 negative instances. With the new dataset, the 15 selected features appear to be still informative for the RF model, but not effective for the SVM and LR models. It is possible that the SVM model in the previous study [17] might have been slightly overfitted by using the 15 selected features.

**Fig. 3:**
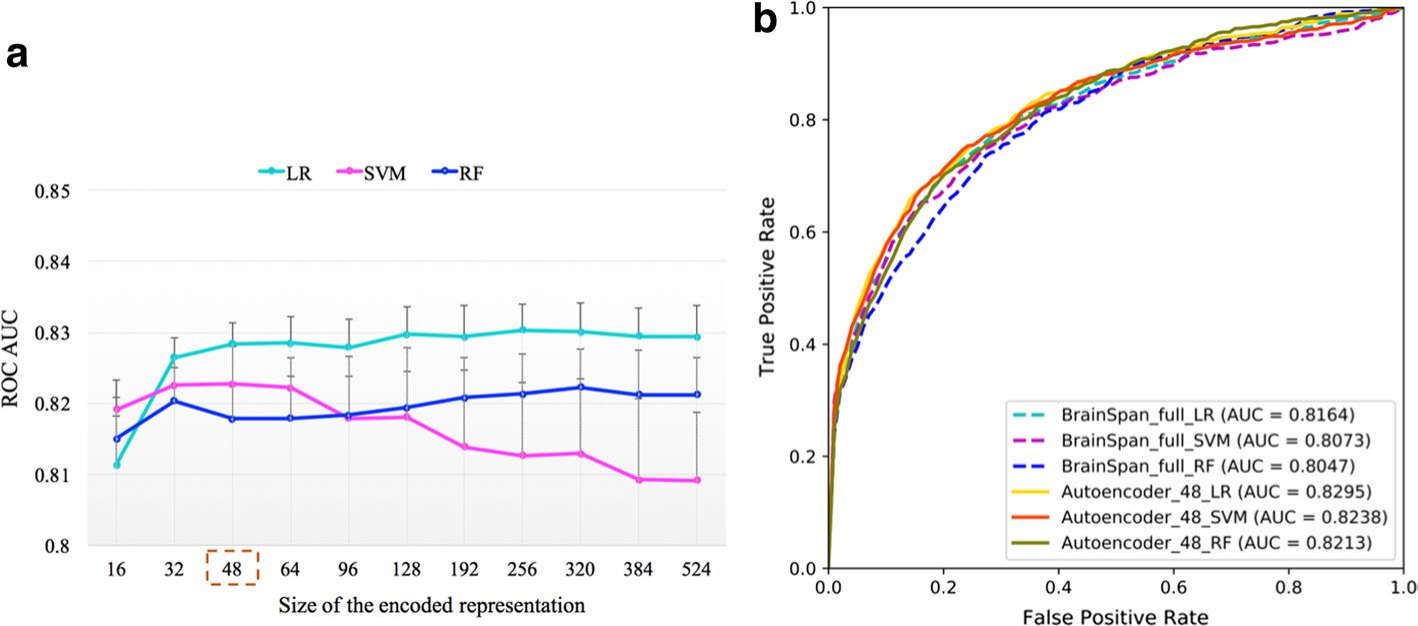
Model performance with unsupervised representation learning of developmental brain gene expression data. **a** Selection of the code dimension size for the autoencoder network. For each dimension size, models were evaluated using the average performance value from fifty repetitions of tenfold cross-validations. Based on the ROC AUC, 48 was selected as the code dimension size for the autoencoder network. **b** ROC curves to compare the models trained using the full set of 524 expression features and the 48 encoded features

Instead of feature selection, unsupervised approaches can be used for feature representation learning and dimensionality reduction. We utilized an autoencoder network to learn a representation of the brain gene expression data. The autoencoder network includes an encoder to learn a representation of the input data, and a decoder to reconstruct the input data from the representation [27, 28]. The representation with a reduced dimensionality can be used for model construction. Various dimension sizes of the representation were tested (Fig. 3a). For each dimension size, models were evaluated using the average performance value from fifty repetitions of tenfold cross-validations. Based on the average ROC AUC, 48 was selected as the encoding size for the autoencoder network. Interestingly, the models trained using the encoded features achieved slightly better performance than the models with the full set of gene expression features (Fig. 3b and Additional file 1: Fig. S1).

We also tested the gene embedding method, Gene2vec [26], which utilized gene co-expression patterns to generate a distributed representation of genes. With Gene2vec, the positive and negative instances in the training dataset for ASD risk gene prediction could be embedded into *n*-dimension vectors for training LR, SVM and RF models (Additional file 1: Table S1). However, the models trained using the gene embedding features did not perform as well as the models with either the full set of expression features or the autoencoder-encoded features (Additional file 1: Fig. S2). Taken together, the autoencoder network outperformed feature selection and gene embedding in this study, as it could efficiently learn a low dimension representation of the developmental brain gene expression data, and improve the model performance for ASD risk gene prediction.

### RNA transcript sequence contains information for ASD risk gene prediction

**Fig. 4:**
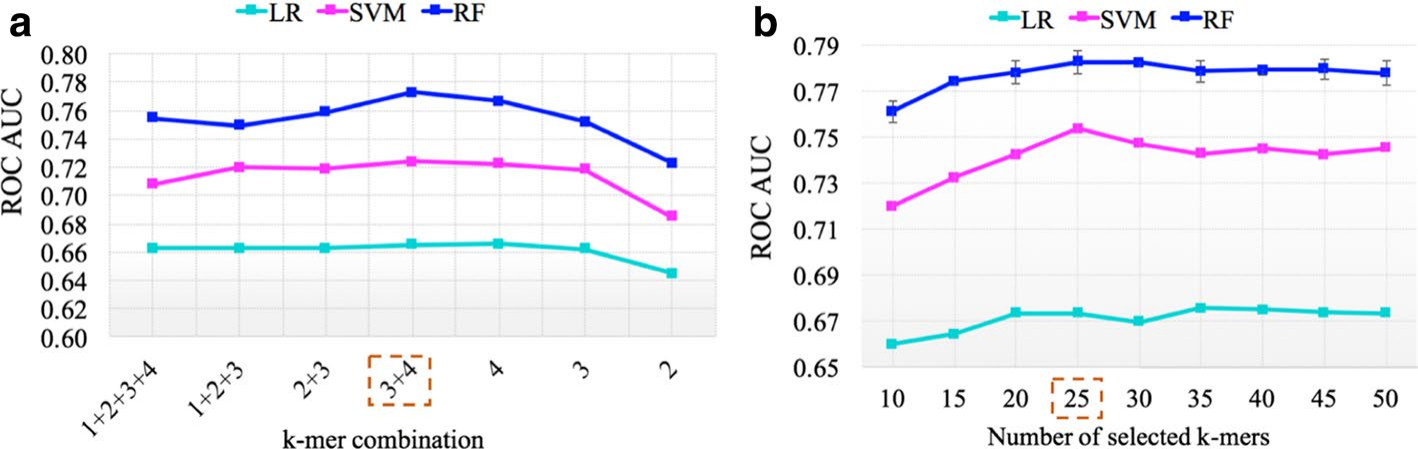
RNA transcript sequence contains relevant information for ASD risk gene prediction. **a** Model performance with different *k*-mer combinations (*k* = 1, 2, 3 or 4). Based on the ROC AUC, 3-mers and 4-mers were selected as the sequence features. **b** Model performance with different numbers of selected *k-*mers. The 3-mers and 4-mers were ranked according to the importance scores provided by the RF model, and the top 25 k-mers were selected for model construction

Besides gene expression data, RNA transcript sequence may also provide relevant features for both protein-coding and non-coding genes. In particular, *k*-mer frequencies, such as the mononucleotide, dinucleotide and trinucleotide compositions, may be used to represent a nucleotide sequence. In this study, we examined the performance of models using different *k*-mer combinations (*k* = 1, 2, 3 or 4). Interestingly, the use of 3-mer and 4-mer nucleotide compositions achieved a ROC AUC of about 0.78, much higher than random guess (ROC AUC = 0.50), suggesting that RNA transcript sequence contains some relevant information for ASD risk gene prediction (Fig. 4a).

Since 3-mer and 4-mer nucleotide compositions gave rise to a relatively large set of 340 features (4^3^ + 4^4^) for the small number of positive instances in the training dataset (604 high-confidence ASD risk genes), we selected a subset of highly ranked *k*-mers based on the importance scores calculated by the RF model. Considering the random initialization of the RF algorithm, we trained the RF mode for ten repetitions, and the average importance scores were used to rank the *k*-mers (Additional file 2). The top 10–50 most important *k*-mers were tested for model construction. As shown in Fig. 4b, the top 25 k-mers achieved the best model performance, and were thus selected as the optimal subset of RNA transcript sequence features. The results suggest that the RF-based feature selection can reduce the dimensionality of *k*-mer sequence features and improve model performance for the three learning algorithms. It is also interesting to note that the SVM and RF models are more accurate than the LR model for the classification of ASD risk genes based on the *k*-mer features.

Next, LR, SVM and RF models were trained using the integrated feature set consisting of 48 autoencoder-derived expression features and 25 RF-selected sequence features. Table 1 gives the average performance measures from 50 repetitions of tenfold cross-validations. Based on the ROC AUC and PR AUC as robust metrics for model evaluation and comparison [29], the LR, SVM and RF models trained using both expression and sequence features showed slightly better performance than the models trained using only the expression features. However, RNA transcript sequence compositions appeared to be less informative than developmental brain gene expression values for ASD risk gene prediction. It is known that there is a large genetic heterogeneity in ASD, involving both locus heterogeneity and allelic heterogeneity [30]. Whether ASD risk genes have common nucleotide sequence features remains unknown. In this study, the model performance indicates that RNA transcript sequence may contain some relevant information for ASD risk gene prediction. Particularly, 17 of the 25 RF-selected *k*-mers have significantly different frequencies (Welch two sample t-test, *p*-value < 0.05) between the RNA transcripts of ASD risk genes and non-ASD disease genes (Additional file 1: Table S2). These *k-*mers may constitute the sequence motifs important for the function and regulation of ASD risk genes.

**Table 1:** Model performance with both developmental brain gene expression features and RNA transcript sequence features

Model	Metric*	Expression features	Expression and sequence features
LR	ROC AUC	0.8289 ± 0.0030	0.8313 ± 0.0024
	PR AUC	0.7096 ± 0.0051	0.7177 ± 0.0044
	Accuracy	0.7615 ± 0.0059	0.7563 ± 0.0054
	Sensitivity	0.7377 ± 0.0071	0.7498 ± 0.0074
	Specificity	0.7719 ± 0.0087	0.7602 ± 0.0087
	MCC	0.4723 ± 0.0095	0.4697 ± 0.0079
SVM	ROC AUC	0.8232 ± 0.0049	0.8217 ± 0.0066
	PR AUC	0.7085 ± 0.0056	0.7159 ± 0.0081
	Accuracy	0.7746 ± 0.0052	0.7745 ± 0.0076
	Sensitivity	0.7111 ± 0.0115	0.7035 ± 0.0156
	Specificity	0.7999 ± 0.0094	0.8023 ± 0.0134
	MCC	0.4826 ± 0.0087	0.4789 ± 0.0123
RF	ROC AUC	0.8187 ± 0.0052	0.8258 ± 0.0053
	PR AUC	0.6908 ± 0.0075	0.7008 ± 0.0113
	Accuracy	0.7767 ± 0.0196	0.7699 ± 0.0188
	Sensitivity	0.6478 ± 0.0468	0.6981 ± 0.0497
	Specificity	0.8271 ± 0.0433	0.7986 ± 0.0401
	MCC	0.4644 ± 0.0207	0.4711 ± 0.0200

*The mean ROC AUC, PR AUC, overall accuracy, sensitivity, specificity and Matthews Correlation Coefficient (MCC) of the models from 50 repetitions of tenfold cross-validations are shown

**Table 2:** The mean percentile rank of known ASD risk genes for the LR, SVM and RF models.

Model	Size of the hypothetical loci		
	101	201	401
LR	80.86	80.94	81.42
SVM	73.94	76.07	77.93
RF	83.85	83.21	83.18

**Table 3:** Top five candidate lncRNAs highly prioritized to be associated with ASD.

Gene ID	Gene symbol	Annotation	Co-expressed protein-coding genes in the BrainSpan dataset (PCC>0.95)*	Traits reported in GWAS Catalog**
ENSG00000229807	XIST	X inactive specific transcript	\	\
ENSG00000240801	AC132217.1	3' overlapping ncRNA IGF2	IGF2	Birth weight
ENSG00000228971	LINC02607	Long intergenic non-protein coding RNA 2607	\	Intelligence, cognitive performance
ENSG00000258283	AC011603.4	Antisense to DDN	DDN	Intelligence, cognitive ability, Bipolar disorder
ENSG00000221857	AC020907.2	Novel transcript	GNPTG, NDRG2, TNFSF12, TNFSF12-TNFSF13	\

*The protein-coding genes co-expressed with the candidate lncRNAs in the BrainSpan dataset are indicated. Pearson Correlation Coefficient (PCC) > 0.95 was used to select the co-expressed gene pairs.

**Traits reported to be associated with the genomic loci from which the candidate lncRNAs are transcribed. Information was retrieved from GWAS Catalog (https://www.ebi.ac.uk/gwas/).

### Model validation for ASD candidate gene prioritization

To further evaluate the models for their capability to predict ASD risk genes, we used a previously described method [17] to prioritize a known ASD risk gene among a list of candidate genes. For each known ASD risk gene, a gene list (called a hypothetical locus) containing the ASD risk gene and its flanking genes was generated. A model was constructed using the training dataset without the target ASD risk gene, and then evaluated by its ability to prioritize the candidate genes in the hypothetical locus. Different numbers of flanking genes (100, 200, and 400) centered around the known ASD risk gene were tested. We assumed that a high-performance model would prioritize the known ASD risk genes in higher percentile ranks.

**Fig. 5:**
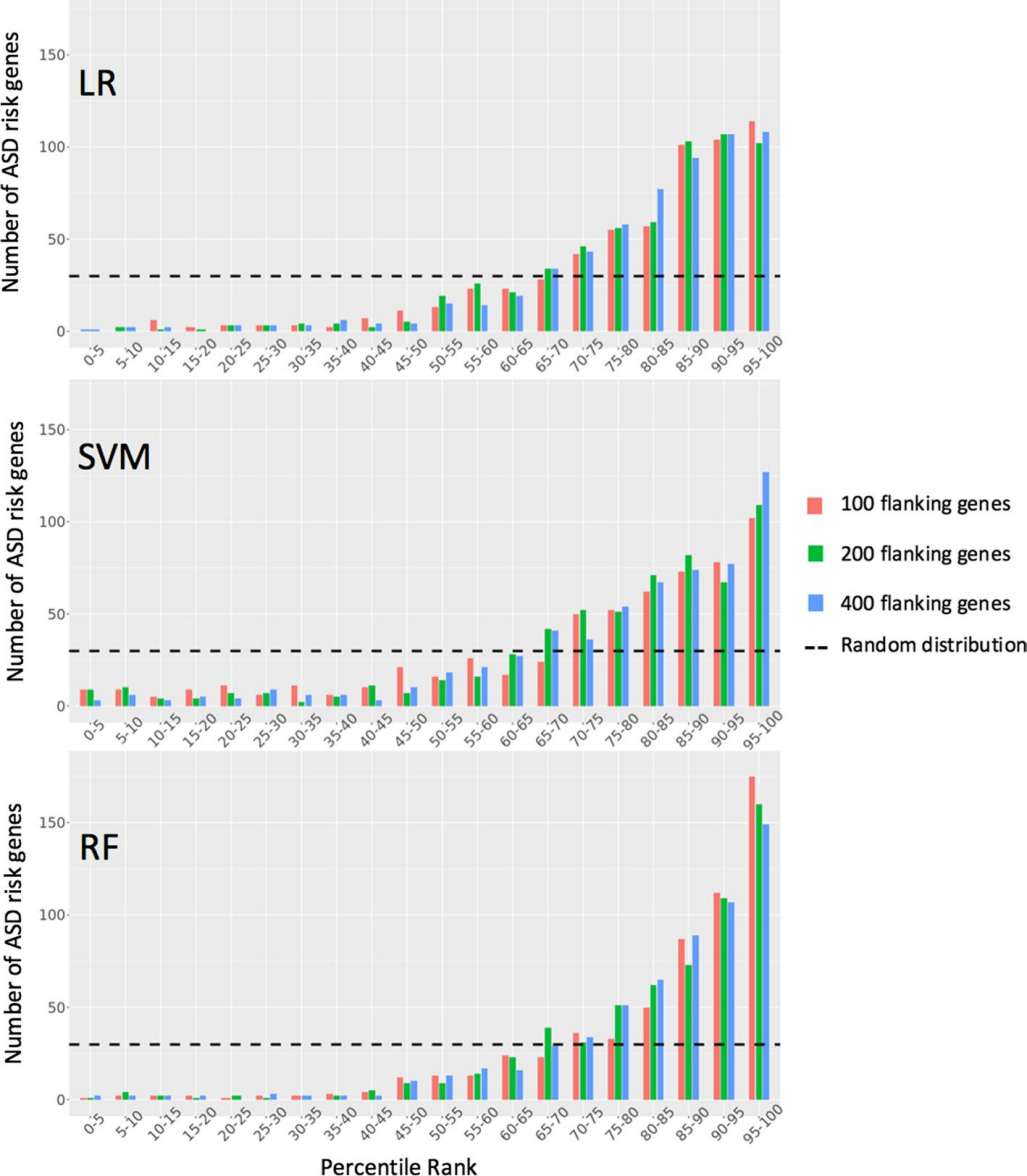
Histogram of ASD risk genes grouped by percentile rank for different lengths of hypothetical loci. For each hypothetical locus centered around the target ASD risk gene, the percentile rank of the known ASD gene was calculated based on the model output. Random distribution is shown in a dashed line (ASD risk genes equally distributed among 20 bins)

As shown in Fig. 5, for all three models, the known ASD risk genes were prioritized mostly above the 85th percentile in the hypothetical loci, and the distributions differed from the random distribution. The number of ASD risk genes increased with the percentile rank, and only a few ASD risk genes were located in the lower percentiles. Moreover, the models showed robust performance for the prioritization of ASD risk genes when the size of candidate gene list was increased from 101 to 401. As shown in Table 2, the mean percentile rank for the LR and SVM models increased with the size of the hypothetical loci, consistent with the assumption that ASD risk genes would remain to be highly prioritized in an expanded gene list. Compared with the LR and SVM models, the RF model appeared to be more accurate with a higher mean percentile rank, and the ASD risk genes classified as positive instances by the RF model were predominantly in the 95th percentile or above (Fig. 5). Therefore, with demonstrated robustness and high predictive performance, our models can provide an efficient way for genome-wide prediction of genes associated with ASD.

### Prediction and prioritization of candidate lncRNAs associated with ASD

LncRNAs have relatively higher levels of expression in the human brain than other body tissues, and have been shown to be involved in neurodevelopment [6, 31]. However, the role of lncRNAs in ASD is still unclear. To identify good candidate lncRNAs, we used the models to prioritize a list of human lncRNAs with detectable expression signals in the BrainSpan dataset. Both the developmental brain gene expression features and RNA transcript sequence features of lncRNAs were utilized for the prediction. Overall, with the probability threshold set at 0.5, 1,124 (11.88%) of 9,463 lncRNAs were predicted to be associated with ASD by at least one of the three models (Additional file 3). Among the 1,124 lncRNAs, 420 (37.37%) were also identified in the previous study [17]. However, only 57 lncRNAs were predicted to be associated with ASD by all three models, and the top five candidate lncRNAs highly prioritized to be associated with ASD are shown in Table 3. XIST is an X-chromosome transcript that initiates X-chromosome inactivation and only expressed in females. It has been suggested that naturally occurring sexually dimorphic processes may modulate the impact of risk variants and contribute to the sex-skewed prevalence of ASD [32]. AC132217.1 and AC011603.4 are transcribed from the genomic loci of the protein-coding genes IGF2 and DDN, respectively, and the two lncRNAs are highly co-expressed with their cognate protein-coding genes in the BrainSpan dataset (Table 3). Interestingly, both IGF2 and DDN have been implicated in the learning-dependent process in mice. IGF2 encodes insulin-like growth factor II, which is a cognitive enhancer and can reverse autism-like phenotypes in mice [33]. DDN encodes a postsynaptic density enriched protein, Dendrin, and the synaptic scaffold protein Kibra modulates learning and memory via binding to Dendrin [34]. Moreover, three of the top five candidate lncRNAs are transcribed from the genomic loci reported to be associated with some autism-related traits, including birth weight, intelligence and cognitive ability, in human GWAS studies.

LncRNAs can act as both *trans-* and *cis-* regulators of gene expression [35, 36], and may be involved in ASD by regulating protein-coding genes. We thus analyzed the relative genomic locations between the candidate lncRNAs and known ASD risk genes. Overall, the candidate lncRNAs showed a similar genomic distribution with ASD risk genes (Additional file 1: Fig. S3). Particularly, 44 of the candidate lncRNAs are transcribed from the genomic loci of ASD risk genes, including 29 antisense lncRNAs of their cognate ASD risk genes (Additional file 3). For instance, the ASD risk gene SATB2 is evolutionarily conserved and encodes a DNA-binding protein involved in transcriptional regulation and chromatin remodeling, and mutations in SATB2 have been reported to be associated with cleft palate, facial dysmorphism and intellectual disability [37]. The lncRNA SATB2-AS1 (ENSG00000225953), consistently predicted to be an ASD-associated lncRNA by all three models, has been shown to *cis*-activate SATB2 transcription via mediating histone H3K4me3 deposition and DNA demethylation of the promoter region of SATB2 in colorectal cancer [38]. The lncRNA RP11-545N8.3 (ENSG00000259125), predicted to be associated with ASD by the LR model, is a conserved antisense lncRNA transcribed from the locus of ASD risk gene LRP1, and can directly bind to chromatin-associated protein HMGB2 to inhibit transcriptional activation of LRP1 [39]. As the knowledge about the functional roles of lncRNAs in ASD is currently limited, the list of lncRNAs predicted to be associated with ASD by the models can provide good targets for further investigation.

## Discussion

In this study, we have demonstrated that machine learning models built using developmental brain gene expression patterns and RNA transcript sequence compositions as features can accurately predict ASD risk genes. The knowledge learnt from known ASD risk genes (protein-coding) can be transferred to lncRNAs for prediction. Using only gene expression profiles during normal brain development as features, an SVM model was previously proposed for ASD risk gene prediction [17]. Our results suggest that RNA transcript sequence also contains some relevant information for ASD risk gene prediction. Compared with the previous study [17], we have developed a more comprehensive approach for model construction, including deep learning techniques and multiple classification algorithms, and obtained accurate models with superior performance for ASD risk gene prediction. Thus, we have utilized the models to predict and prioritize a list of candidate lncRNAs that may be involved in ASD. Notably, some highly prioritized candidate lncRNAs are co-expressed with known ASD risk genes (protein-coding) during brain development. Since it is still unclear whether and how lncRNAs contribute to ASD risk, our findings can provide valuable information for functional characterization of the candidate lncRNAs associated with ASD.

One limitation of this study is that the models make predictions highly based on the gene expression profiles in the BrainSpan dataset, which has been collected from postmortem normal brain tissue samples, not autistic brain samples. This drawback of the current training dataset may limit the model performance. We hope that high-quality gene expression profiles of autistic brains can become publicly available in the future to facilitate the further development of accurate models for ASD risk gene prediction.

## Conclusions

In sum, we have developed an efficient machine learning approach for genome-wide prediction of candidate lncRNAs associated with ASD. Both developmental brain gene expression data and RNA transcript sequence were utilized as features to construct accurate and robust models with different machine learning algorithms. To reduce input dimensionality and avoid model overfitting, we tested an autoencoder network for representation learning of gene expression data and a random-forest-based method for sequence feature selection. The high predictive performance of the models was demonstrated by their capability to accurately predict ASD risk genes in hypothetical loci. We then applied the models to predict and prioritize a list of candidate lncRNAs for further investigation.

## Methods

### Datasets

In this study, the positive instances included 604 high-confidence ASD risk genes collected from the Simons Foundation Autism Research Initiative (SFARI) database (https://www.sfari.org/resource/sfari-gene/) in the gene category S/1/2/3, and the negative instances were 1,594 non-ASD disease genes compiled by a previous study [17] and curated for no implication in ASD.

### Gene expression and RNA sequence features

Gene expression profiles were obtained from the BrainSpan Atlas of the Developing Human Brain (https://www.brainspan.org). BrainSpan provides a developmental transcriptome dataset consisting of 524 samples with developmental time points ranging from 8 weeks postconception to 40 years old from 26 brain structures, and the gene expression values are represented in reads per kilobase of transcript per million mapped reads (RPKM) [40]. A log_2_(RPKM + 1) transformation of the gene expression values was performed as described previously [17].

To derive nucleotide sequence features, protein-coding transcript sequences were extracted from the GENCODE FASTA file (GRCh38) (https://www.gencodegenes.org/human/). For each RNA sequence, the frequencies of *k*-mers were calculated and normalized by the sequence length. The min–max transformation was performed to normalize the *k*-mer features as well as the combined set of gene expression and sequence features.

### Autoencoder

To reduce the dimensionality of gene expression features, an autoencoder network was implemented (Fig. 1). The network included an encoder layer to transform the high-dimensional data, ${x}_{in}$, into a low-dimensional code, ${x}_{code}$, and a decoder layer to recover the data from the code:

1 $$f^{{(ReLU)}} x_{i} n = x_{{code}}$$

2 $$f^{{(ReLU)}} x_{code} n = x_{{in}}$$

The nonlinear function, rectified linear unit (ReLU), was used in both the encoder layer and the decoder layer. ReLU outputs a positive value and 0 otherwise (negative values clamped to 0). The dimension size of ${x}_{code}$ was tested from 16 to 524. The mean squared error (MSE) and the root mean square prop (RMSprop) were used as the loss function and the optimizer, respectively. The autoencoder network was trained for 100 epochs with a batch size of 64.

### Embedding

To reduce the dimensionality of gene expression features, we also tested an embedding technique, Gene2vec, which could utilize gene co-expression patterns to generate a distributed representation of each gene [26]. First, gene co-expression was measured by Pearson Correlation Coefficient (PCC) using the BrainSpan dataset. Second, co-expressed gene pairs were selected and served as the training data for Gene2vec to learn a *n*-dimension vector representation for each gene in the BrainSpan dataset. Third, the positive and negative instances in the training dataset for ASD risk gene prediction were represented by the *n*-dimension vectors to train LR, SVM and RF models. For Gene2vec, a number of parameters were tested. PCC > 0.5 and PCC > 0.9 were tested to select co-expressed gene pairs. As the number of iterations (*i*) and dimensionality (*n*) of the embedding were the two major hyper-parameters for Gene2vec [26], *i* was tested in the range from 1 to 10, and *n* was tested in the range from 50 to 300.

### Model construction

Different machine learning algorithms, including logistic regression (LR), support vector machine (SVM) and random forest (RF), were tested, and the Scikit-learn Python library [41] was used for model construction. Since the training dataset had a 1:2.6391 ratio of positive to negative instances, the class weight for negative/positive was set to 1/2.6391. Various training parameters were tuned for higher model performance (Additional file 1: Table S3).

### Model evaluation

Models were trained and evaluated using a tenfold cross-validation strategy. The dataset with 604 positive instances and 1,594 negative instances was randomly and equally divided into ten subsets, and the 1:2.6391 ratio of positive to negative instances was maintained in each subset. Models were evaluated by holding out each subset in turn for testing and training on the remaining nine subsets. The average of the ten evaluation results was taken as the final model performance. The following metrics were used for model evaluation:

3 $${\text{Accuracy}} = \frac{{TP + TN}}{{TP + TN + FP + FN}}$$

4 $${\text{Sensitivity}} = \frac{{TP}}{{TP~ + ~FN}}$$

5 $${\text{Specificity}} = \frac{{TN}}{{TN + FP}}$$

6 $${\text{Precision}} = \frac{{TP}}{{TP + FP}}$$

7 $${\text{MCC}} = \frac{{TP~ \times ~TN - FP~ \times ~FN}}{{\sqrt {\left( {TP + FP} \right)\left( {TP + FN} \right)\left( {TN + FP} \right)\left( {TN + FN} \right)} }}$$

In the above formulas, TP is the number of true positives; TN is the number of true negatives; FP is the number of false positives; FN is the number of false negatives. The Matthews correlation coefficient (MCC) measures the correlation between predictions and actual labels. The receiver operating characteristic curve (ROC) is the plot of the true positive rate against the false positive rate with different model output thresholds. A perfect model has an area under the ROC (ROC AUC) of 1.0, whereas random guess has an ROC AUC of 0.5. The precision-recall (PR) curve is the plot of precision against recall (sensitivity) with different model output thresholds. A perfect model has an area under the PR (PR AUC) of 1.0, whereas random guess has an PR AUC of 0.5.

### Model validation using hypothetical loci

To examine the capability of models to prioritize candidate ASD risk genes, we used the method described previously [17]. Briefly, for each known ASD risk gene in the training dataset, $N$ neighboring genes ($N$ = 101, 201 and 401) centered on the ASD risk gene on the same chromosome were extracted from the GENCODE GRCh38 comprehensive gene annotation file (https://www.gencodegenes.org/human/) to create a hypothetical locus. A model was constructed using the training dataset without the target ASD risk gene, and then used to predict and prioritize the candidate genes in the hypothetical locus. Model performance was evaluated by the percentile rank calculated for the target ASD risk gene within the corresponding hypothetical locus:

8 $${\text{Percentile rank}} = \frac{L}{N}\, \times \,{\text{1}}00\%$$

Here, $L$ represents the number of genes with probabilities less than the target ASD risk gene, $N$ is the number of all genes within the hypothetical locus.

## Supplementary information

**Additional file 1**. Supplementary figures and tables.

**Additional file 2**. The average importance score calculated by the random forest model for each of the 320 k-mer features.

**Additional file 3**. ASD-associated candidate lncRNAs overlapping with the genomic loci of the known ASD risk genes.

## Data Availability

Datasets and models are available in the GitHub repository (https://github.com/BioDataLearning/LncAutism).

## Abbreviations

ASD: Autism spectrum disorder
CNV: Copy number variation
DDN: Dendrin
FDR: False discovery rate
FN: False negative
FP: False positive
GNPTG: N-acetylglucosamine-1-phosphate transferase
GWAS: Genome-wide association study
**IGF2**: Insulin like growth factor 2
**LncRNAs**: Long non-coding RNAs
**LR**: Logistic regression
**MCC**: Matthews correlation coefficient
**miRNA**: MicroRNA
MRI: Magnetic resonance image
**MSE**: Mean squared error
**NDRG2**: N-Myc downstream-regulated gene 2 protein
**PCC**: Pearson correlation coefficient
**PR AUC**: Area under the precision-recall curve
**ReLU**: Rectified linear unit
**RF**: Random forest
**RMSprop**: Root mean square prop
**RPKM**: Reads per kilobase of transcript per million mapped reads
**ROC AUC**: Area under the receiver operator characteristic curve
SVM: Support vector machine
SFARI: Simons foundation autism research initiative
**TP**: True positive
**TN**: True negative
**TNFSF12**: Tumor necrosis factor (ligand) superfamily, member 12
TNFSF13: Tumor necrosis factor (ligand) superfamily, member 13
**WES**: Whole exome sequencing
**XIST**: X-inactive specific transcript
