# Supplementary material for: Prediction and prioritization of autism-associated long non-coding RNAs using gene expression and sequence features
Source: BMC Bioinformatics. 2020 Nov 7;21:505. doi: 10.1186/s12859-020-03843-5 (PMC7648398; doi:10.1186/s12859-020-03843-5)
Supplement: Supplementary file 1 — Additional file 1. Supplementary figures and tables. [file 12859_2020_3843_MOESM1_ESM.pdf]

Supplementary Figures and Tables for

Prediction and prioritization of autism-associated long non-coding RNAs using gene expression and sequence features

Jun Wang[1] and Liangjiang Wang[1,2,*]

[1]Department of Genetics and Biochemistry, [2]Center for Human Genetics, Clemson University, Clemson, South Carolina, 29634, USA.

*To whom correspondence should be addressed. Tel: (+1) 864-656-0733; Fax: (+1) 864-656-0393; Email: liangjw@clemson.edu

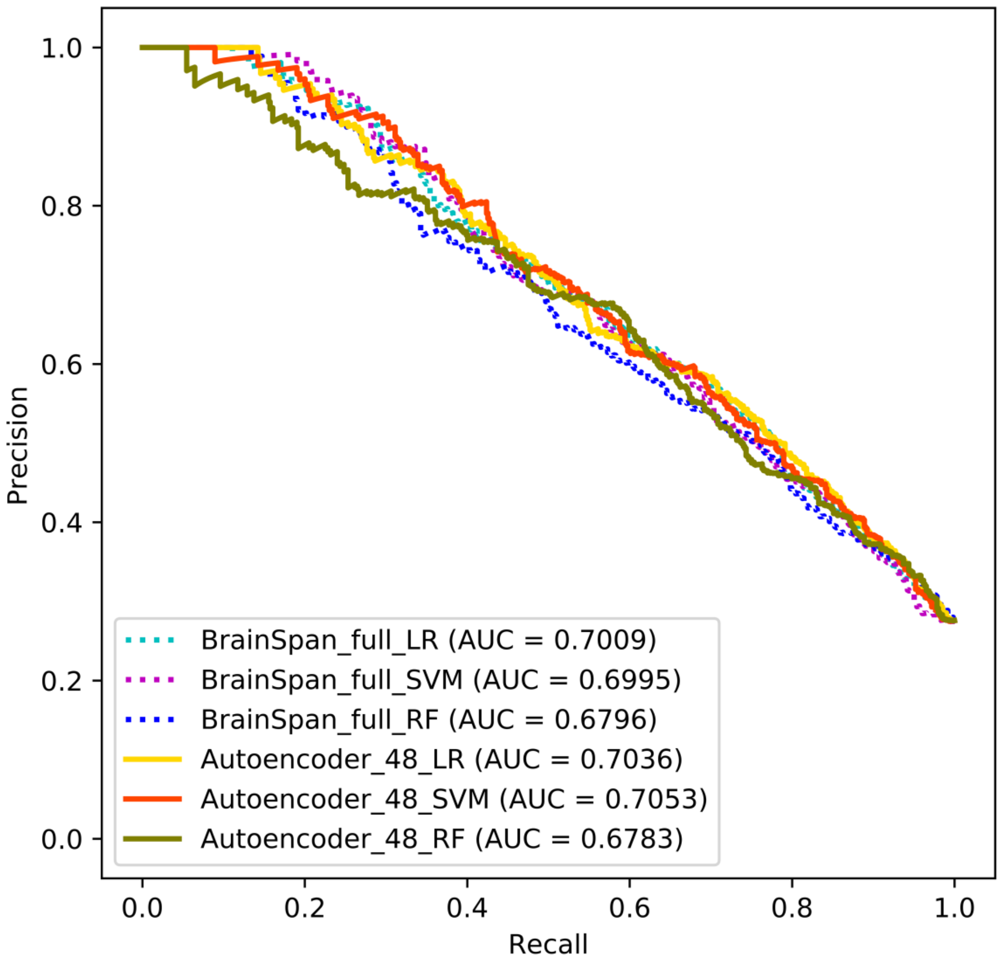

Fig. S1. PR curves to compare the models trained with the full set of 524 expression features and the 48 autoencoder-derived features.

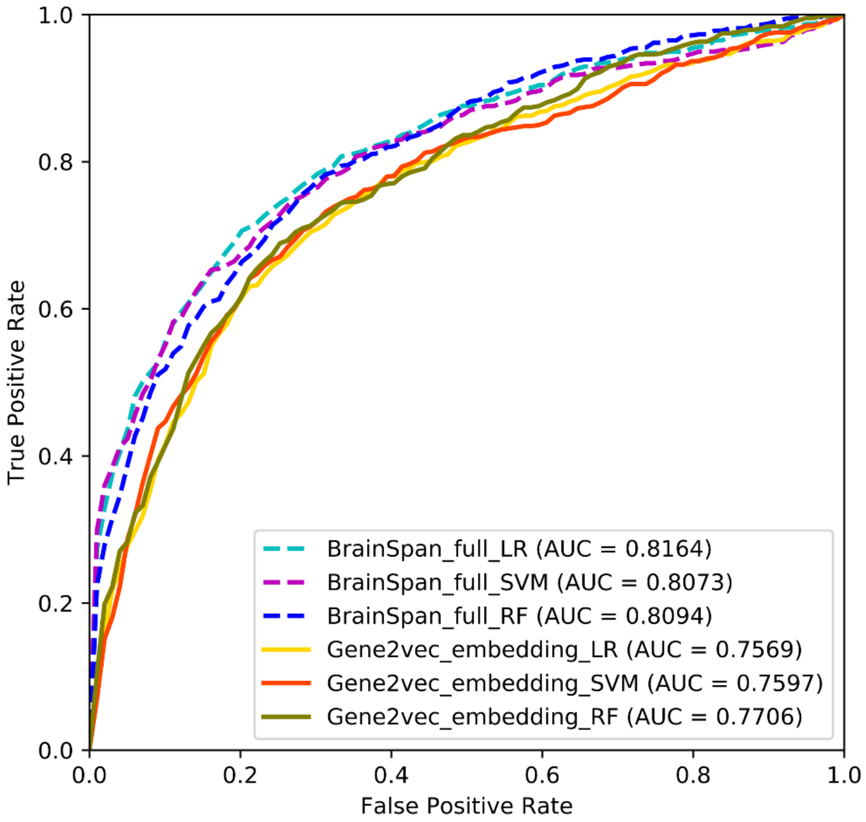

Fig. S2. ROC curves to compare the models trained with the full set of 524 expression features and the Gene2vec-embedded features. According to the ROC AUC of the three models using tenfold cross-validations, PCC > 0.5 was used to select co-expressed gene pairs in the BrainSpan dataset, and the dimension of 300 at iteration 4 was found to produce gene embedding with the best performance of LR, SVM and RF models.

LR

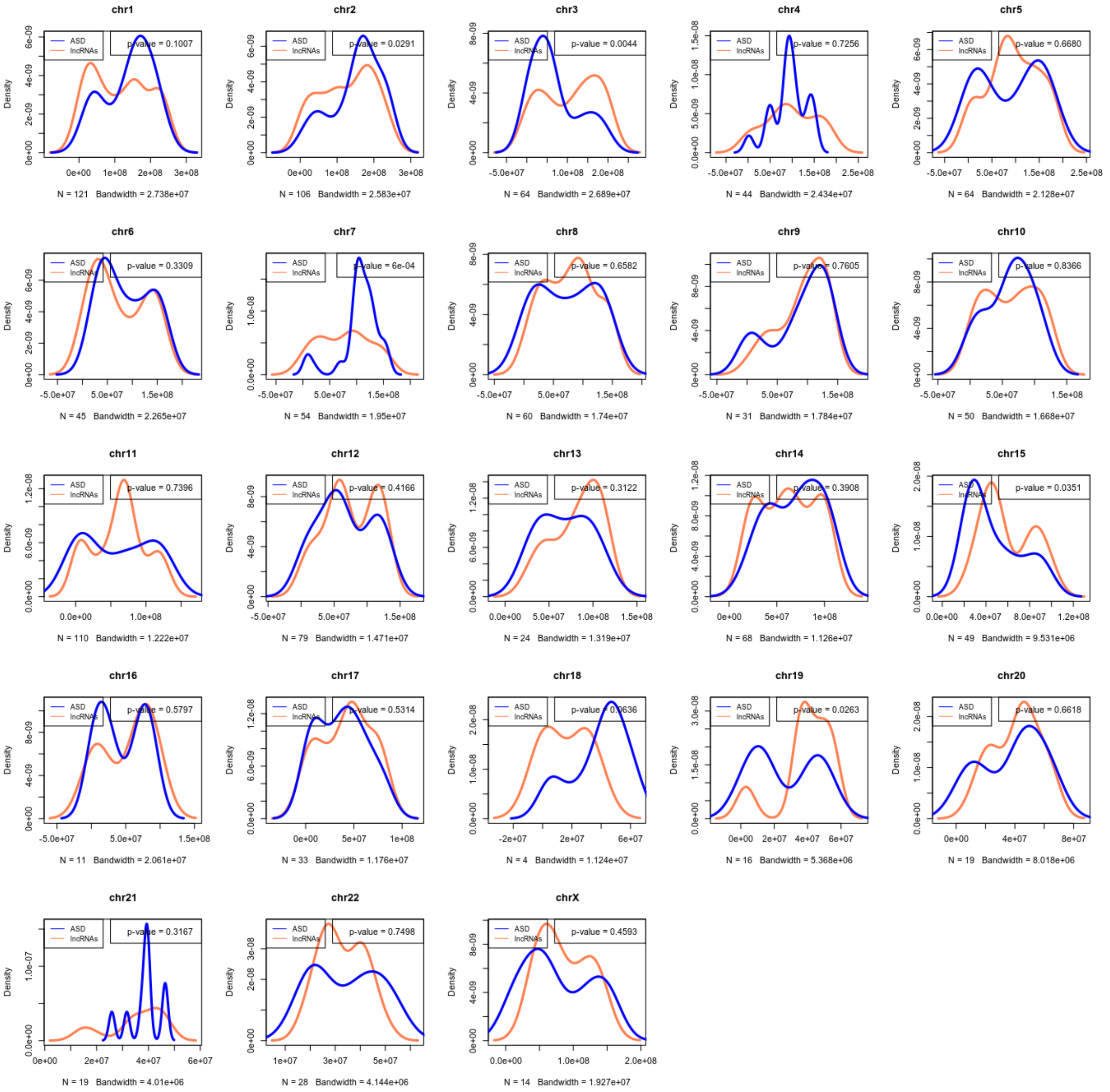
chr1
ASD
lncRNAs
p-value = 0.1007
N = 121 Bandwidth = 2.738e+07
chr2
p-value = 0.0291
N = 106 Bandwidth = 2.583e+07
chr3
p-value = 0.0044
N = 64 Bandwidth = 2.689e+07
chr4
p-value = 0.7256
N = 44 Bandwidth = 2.434e+07
chr5
p-value = 0.6680
N = 64 Bandwidth = 2.128e+07
chr6
p-value = 0.3309
N = 45 Bandwidth = 2.265e+07
chr7
p-value = 6e-04
N = 54 Bandwidth = 1.95e+07
chr8
p-value = 0.6582
N = 60 Bandwidth = 1.74e+07
chr9
p-value = 0.7605
N = 31 Bandwidth = 1.784e+07
chr10
p-value = 0.8366
N = 50 Bandwidth = 1.668e+07
chr11
p-value = 0.7396
N = 110 Bandwidth = 1.222e+07
chr12
p-value = 0.4166
N = 79 Bandwidth = 1.471e+07
chr13
p-value = 0.3122
N = 24 Bandwidth = 1.319e+07
chr14
p-value = 0.3908
N = 68 Bandwidth = 1.126e+07
chr15
p-value = 0.0351
N = 49 Bandwidth = 9.531e+06
chr16
p-value = 0.5797
N = 11 Bandwidth = 2.061e+07
chr17
p-value = 0.5314
N = 33 Bandwidth = 1.176e+07
chr18
p-value = 0.0636
N = 4 Bandwidth = 1.124e+07
chr19
p-value = 0.0263
N = 16 Bandwidth = 5.368e+06
chr20
p-value = 0.6618
N = 19 Bandwidth = 8.018e+06
chr21
p-value = 0.3167
N = 19 Bandwidth = 4.01e+06
chr22
p-value = 0.7498
N = 28 Bandwidth = 4.144e+06
chrX
p-value = 0.4593
N = 14 Bandwidth = 1.927e+07
Density

SVM

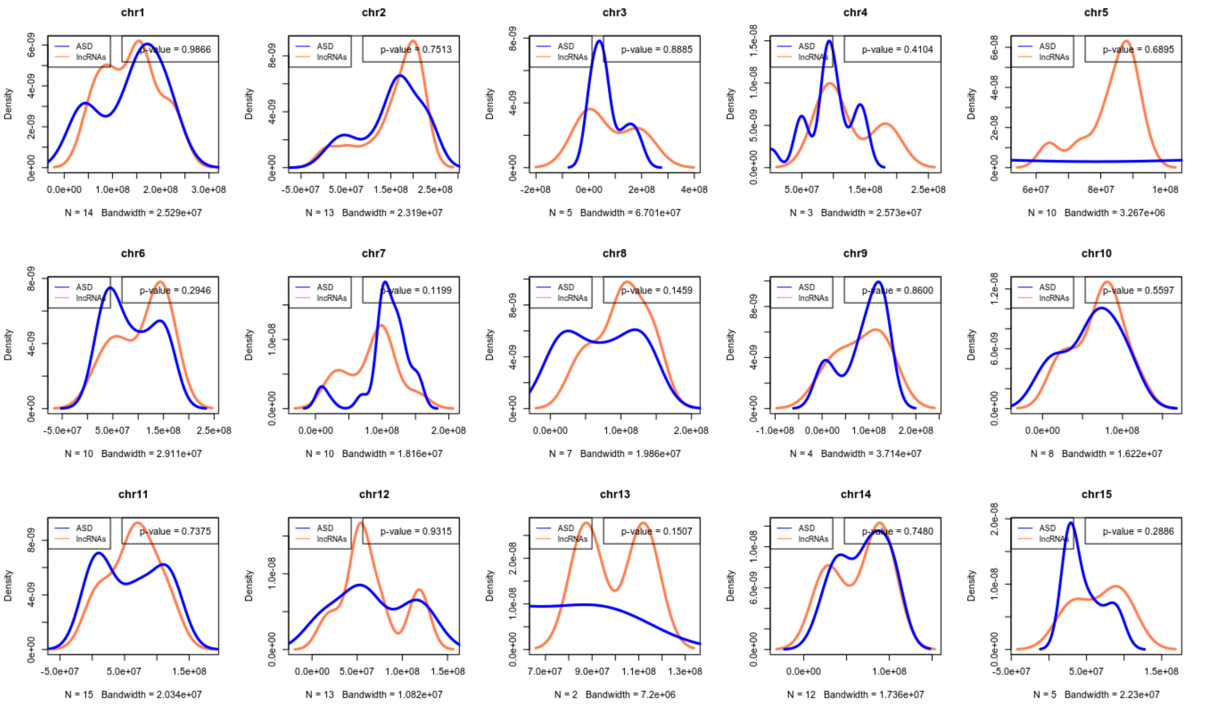

RF

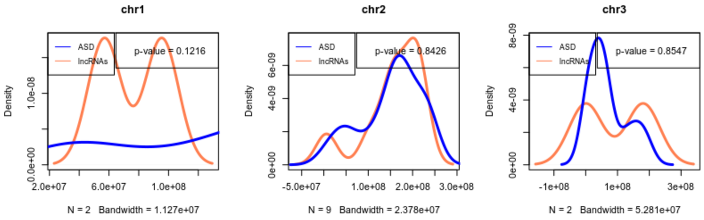

Fig. S3. Genomic distributions of ASD-associated candidate lncRNAs and the known ASD risk genes. Density plot based on the gene starting site was performed. Two-sample Kolmogorov-Smirnov (KS) test was used to examine the statistical significance of similarity between distributions. The *p*-value ranges from 0 to 1, with 0 for no similarity between the genomic distributions of candidate lncRNAs and ASD risk genes, and 1 for the same distributions.

Table S1. Performance of models using the Gene2vec-embedded features*.

PCC	Model	Dimension	Number of Iterations									
			1	2	3	4	5	6	7	8	9	10
0.5	LR	50	0.74	0.75	0.76	0.76	0.76	0.76	0.76	0.76	0.76	0.76
		100	0.74	0.76	0.76	0.76	0.76	0.76	0.76	0.76	0.76	0.76
		200	0.74	0.76	0.76	0.76	0.75	0.76	0.75	0.75	0.75	0.75
		300	0.74	0.76	0.76	0.76	0.76	0.76	0.76	0.75	0.75	0.75
	SVM	50	0.75	0.75	0.76	0.76	0.76	0.76	0.77	0.77	0.76	0.77
		100	0.75	0.76	0.76	0.77	0.77	0.76	0.76	0.76	0.76	0.77
		200	0.76	0.73	0.73	0.76	0.75	0.76	0.76	0.77	0.76	0.75
		300	0.76	0.75	0.75	0.76	0.76	0.78	0.76	0.76	0.76	0.76
	RF	50	0.76	0.78	0.77	0.78	0.77	0.77	0.78	0.77	0.79	0.77
		100	0.77	0.77	0.80	0.78	0.78	0.80	0.78	0.80	0.80	0.80
		200	0.77	0.79	0.78	0.78	0.78	0.80	0.78	0.78	0.78	0.78
		300	0.76	0.77	0.77	0.80	0.78	0.77	0.78	0.79	0.80	0.78
0.9	LR	50	0.51	0.51	0.51	0.53	0.55	0.55	0.56	0.57	0.57	0.58
		100	0.51	0.53	0.53	0.53	0.56	0.56	0.56	0.56	0.56	0.56
		200	0.51	0.52	0.53	0.57	0.59	0.60	0.60	0.60	0.60	0.59
		300	0.54	0.54	0.55	0.56	0.56	0.56	0.57	0.58	0.58	0.58
	SVM	50	0.57	0.56	0.56	0.53	0.55	0.56	0.56	0.56	0.56	0.56
		100	0.60	0.61	0.62	0.55	0.56	0.56	0.57	0.58	0.58	0.58
		200	0.59	0.60	0.60	0.56	0.59	0.58	0.59	0.59	0.58	0.58
		300	0.62	0.63	0.64	0.63	0.58	0.58	0.58	0.58	0.58	0.58
	RF	50	0.59	0.59	0.59	0.63	0.61	0.63	0.62	0.64	0.63	0.63
		100	0.60	0.60	0.64	0.65	0.64	0.65	0.65	0.65	0.64	0.65
		200	0.60	0.61	0.64	0.64	0.64	0.67	0.65	0.66	0.66	0.67
		300	0.62	0.64	0.66	0.66	0.65	0.66	0.66	0.65	0.66	0.65

***** The ROC AUC values of the models from tenfold cross-validations are shown. For the Gene2vec model, various hyper-parameters, Pearson Correlation Coefficient (PCC) and number of iterations were tested to generate the best Gene2vec-embedded features to train LR, SVM and RF models.

Table S2. Frequency comparisons for the 25 *k*-mer features in the positive and negative instances.

***k*-mers**	***p*-value***	**Higher frequency in the positive (P) or negative (N) instances**
CGTT	3.08E-07	P
TGGG	1.69E-19	N
TGG	3.45E-23	N
CTGG	1.81E-25	N
CGCG	0.238453114	\
CTCA	2.08E-08	N
GTCA	0.002501482	P
CGAC	0.696533812	\
TGAG	2.33E-11	N
CTG	2.58E-19	N
ACCT	1.44E-10	N
TGGC	1.51E-16	N
CCTG	8.29E-17	N
ATCT	0.50649693	\
AAGG	0.033015097	N
CGTA	0.000189088	P
CGTC	0.835369262	\
CCGT	0.969627512	\
TTCG	0.027247256	P
AGCG	0.992167296	\
GGGT	2.61E-11	N
ATCG	0.412519657	\
ACA	8.01E-09	P
GCGT	0.01094848	N
GCGG	0.323093951	\

***** The *p*-value was calculated using the Welch two sample t-test.

Table S3. Training parameters tuned for the best mode performance.

Model	Parameter symbol	Parameter description	Testing range	Determined parameter
LR	C	Inverse of regularization strength	0.1-2	1.660055524
	n_jobs	Number of CPU cores used	1-5	2
	max_iter	Maximum iteration number for the solvers to converge	256	256
	solver	Algorithm	'lbfgs'	'lbfgs'
	penalty	The norm used in the penalization	'l1', 'l2'	l2
SVM	kernel	Kernel type to be used	'rbf'	'rbf'
	C	Penalty parameter of the error	2-526	517.881968350
	gamma	Kernel coefficient	0.001-0.1	0.069482974
RF	n_estimators	Number of trees	32-500	128
	criterion	Function to control split quality	'gini', 'entropy'	'gini'
	max_depth	Maximum depth of the tree	5-12	5
	min_samples_split	Minimum number of samples to split an internal node	2-8	4
	max_features	Function to determine the feature numbers for best splits	'log2', 'sqrt'	'log2'
